# How to do quantile normalization correctly for gene expression data analyses

**DOI:** 10.1038/s41598-020-72664-6

**Published:** 2020-09-23

**Authors:** Yaxing Zhao, Limsoon Wong, Wilson Wen Bin Goh

**Affiliations:** 1 School of Pharmaceutical Science and Technology, Tianjin University, Tianjin, China; 2 Department of Computer Science, National University of Singapore, Singapore, Singapore; 3 Department of Pathology, National University of Singapore, Singapore, Singapore; 4 School of Biological Sciences, Nanyang Technological University, Singapore, Singapore

**Keywords:** Biological techniques, Computational biology and bioinformatics

## Abstract

Quantile normalization is an important normalization technique commonly used in high-dimensional data analysis. However, it is susceptible to class-effect proportion effects (the proportion of class-correlated variables in a dataset) and batch effects (the presence of potentially confounding technical variation) when applied blindly on whole data sets, resulting in higher false-positive and false-negative rates. We evaluate five strategies for performing quantile normalization, and demonstrate that good performance in terms of batch-effect correction and statistical feature selection can be readily achieved by first splitting data by sample class-labels before performing quantile normalization independently on each split (“Class-specific”). Via simulations with both real and simulated batch effects, we demonstrate that the “Class-specific” strategy (and others relying on similar principles) readily outperform whole-data quantile normalization, and is robust-preserving useful signals even during the combined analysis of separately-normalized datasets. Quantile normalization is a commonly used procedure. But when carelessly applied on whole datasets without first considering class-effect proportion and batch effects, can result in poor performance. If quantile normalization must be used, then we recommend using the “Class-specific” strategy.

## Introduction

High-throughput technologies such as genomics, transcriptomics and proteomics (-omics) are powerful assays for profiling the expressional state of cellular tissues. This facilitates comparative analysis, providing an in-depth perspective on differential change, with the abductive implication that differential change is correlated with cause^1^.

However, -omics platforms are susceptible to various sources of technical variation such as general noise^2^ and batch effects^3,4^. These technical variations cause the overall measurement distributions of samples (across all genes or proteins) to shift differently, obstructing cross comparability between tissues.

Dealing with technical variation requires an analytical intervention known as normalization. Normalization is not one technique, but rather, a body of techniques, with each having optimal usage requirements. Normalization techniques also make varied assumptions about data distribution. For example, some such as the quantile normalization, assumes all samples have similar distribution regardless of sample class. However, this assumption only holds true when small numbers of genes/proteins are dysregulated.

The purpose of normalization is to eliminate or minimize technical variability. A general strategy, common to many normalization techniques, is to re-distribute signal intensities across all samples such that they now all have the same distribution (e.g. same mean and/or standard deviation). Common examples of normalization techniques include linear scaling (also known as min–max scaling), Z-normalization, and rank-scaling (also known as linear interpolation). Specialized approaches for removing batch effects (a form of technical variation) such as ComBat^5^ and Surrogate Variable Analysis^6^ may also be considered as subtypes of normalization techniques.

It is widely known that normalization techniques are imperfect, and even error generating, especially when the data does not meet the assumptions of the normalization technique^7,8^. In Wu et al.^8^, they examined the distortions produced when cancerous cells (with highly active expressional programmes) are cross-normalized to the same baseline as normal cells. The distortions produced include false effects (false positives), effect-size reduction, and masking of true effects (false negatives). Wang et al.^7^, also demonstrated that different normalization techniques result in different differential gene sets. In both papers, while they attribute the distortions to an imbalance of genomic count (as cancer cells tend to have highly up-regulated expressional programmes), they and in other related studies^7–10^ do not offer tangible mitigating measures. And so, while the analyst (or biologist) is now aware that normalization is imperfect, they are left none-the-wiser on best practices, or at least, how to perform normalization properly.

**Figure 1:**
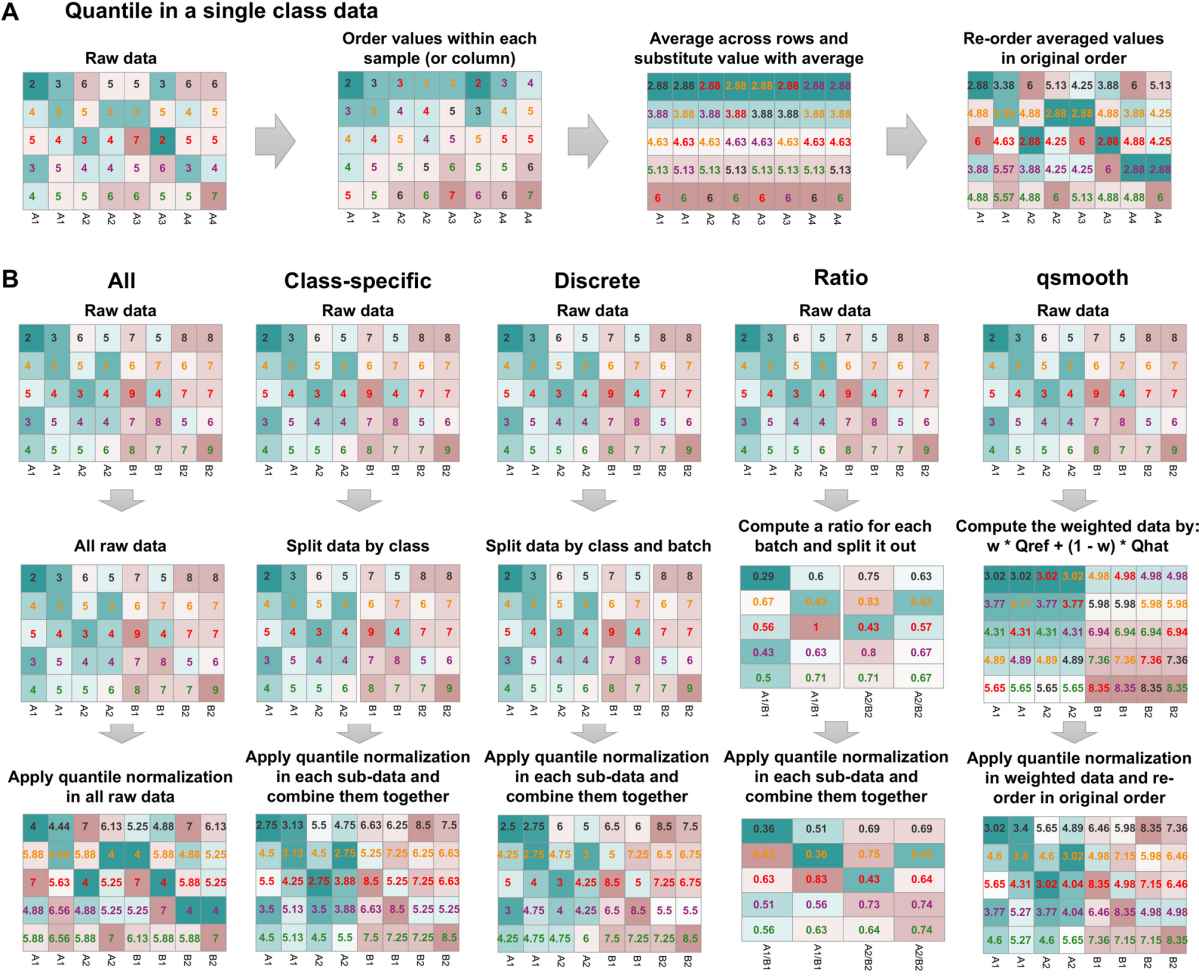
(**A**) The basic steps of quantile normalization, (**B**) 5 different sub-flavors of quantile normalization. All refers to standard quantile normalization, the other sub-flavors include Class-specific, Discrete, Ratio and qsmooth (Qref: reference quantile (row mean). Qhat: linear model fit at each quantile. w: a weight at every quantile that compares the variability between groups relative to within groups).

Since many normalization techniques exist, a one-size-fits-all recommendation study is unfeasible. Instead, we focus on how to use one popular normalization technique properly, the Quantile normalization (QN) method. QN is extremely popular and produces very well-aligned distributions such that QN-normalized samples all have the same distributions^11^ (Fig. 1A). It sees widespread use, constituting a standard part of analysis pipelines for high-throughput analysis^12^.

QN was originally developed for gene expression microarrays^13^ but is now used across almost any kind of high-dimensional/-throughput -omics platform including RNA-sequencing^14^ and proteomics^9^. A particular danger in the use of QN is that lay analysts are easily misled by the rather “perfect-looking” post-normalization results: QN-normalized samples look deceptively similar, even if the underlying classes are in fact, very different. Furthermore, as with Wang et al.’s observations, QN can obliterate true signals and generate false signals during data analysis^7^.

Here, we examine five different (sub)strategies based on the QN-some not necessarily correct, but still shown here so that its deficiencies are made known-for performing analysis (Fig. 1B). These strategies are “All”, the quintessential QN approach that is performed on the whole dataset; “Class-specific”, which splits data first by class, on which QN is then performed separately on each split and then combining the separately-QN normalized splits; “Discrete”, which is similar to “Class-specific”, but goes further to split data by both technical batch and class (i.e. QN is applied separately to each sub-dataset of the same batch and class); “Ratio”, which produces a matrix of ratios by comparing inter-class samples; and qsmooth, which compares inter-class variability against within-class variability to introduce weights in the QN-normalized matrix (see “Materials and methods” section for details).

To determine the best strategy (if one does indeed exist), we benchmark on an assortment of available proteomics data (proteomics data primarily, as an instance of high-throughput biological data) and consider both natural and simulated batch effects across different levels of class-effect proportion (CEP), which is the proportion of differential proteins amongst all measured proteins, between two sample classes. We also evaluate which QN-strategy is robust in situations where datasets that were previously normalized on separate occasions, are merged. This procedure is especially valuable in practical applications such as boosting mega-analysis where datasets are combined from various independently-derived sources to boost statistical power^15^.

## Materials and methods

### Quantile normalization procedure

The quantile normalization (QN) procedure is simple (Fig. 1A): it involves first ranking the gene of each sample by magnitude, calculating the average value for genes occupying the same rank, and then substituting the values of all genes occupying that particular rank with this average value. The next step is to reorder the genes of each sample in their original order. This series of steps characterizes quantile normalization, and is the basic procedure underlying the various (sub)strategies described in Fig. 1B.

Amongst QN-strategies (Fig. 1B), ‘All’ normalizes data as one complete set (irrespective of class and batch factors; a batch factor is a categorization of data by technical effects, also known as batch effects).

The “Class-specific” strategy splits data by phenotype classes first where the classes are then quantile-normalized independently. The normalized splits are then recombined into one dataset. This design is meant to counteract false positives/negatives caused by averaging out sample classes with highly different expressional profiles (e.g. cancer and normal tissues).

The “Discrete” strategy takes the “Class-specific” approach further, and also accounts for the batch factor. Each split (by class and batch) are then quantile-normalized separately, and then recombined into one dataset.

The “Ratio” strategy involves generating a matrix of ratios, obtained by arbitrarily comparing samples from one class against another sample belonging to the other class. Suppose we have a sample S1,A from class A, with proteins 1–1000, and another (paired) sample S1,B from class B, with proteins 1–1000. We may calculate an expression vector B/A, such that for each protein i, its respective ratio is Exp_S1,B,i_/Exp_S1,A,i_ (where Exp is the protein expression value). Note that we only compare samples from the same technical batch. This “ratio-ed” matrix still preserves the batch factors while class effect is now effectively, a fold change.

Unlike the other strategies discussed earlier, the “qsmooth” strategy is a generalized version of QN which preserves global differences in distributions corresponding to different biological conditions^20^. qsmooth computes a weight at every quantile comparing the variability between groups relative to within groups. The weight shrinks the group-level quantile normalized data towards the overall reference quantiles if variability between groups is sufficiently smaller than the variability within groups.

### Overall evaluation strategy

**Figure 2:**
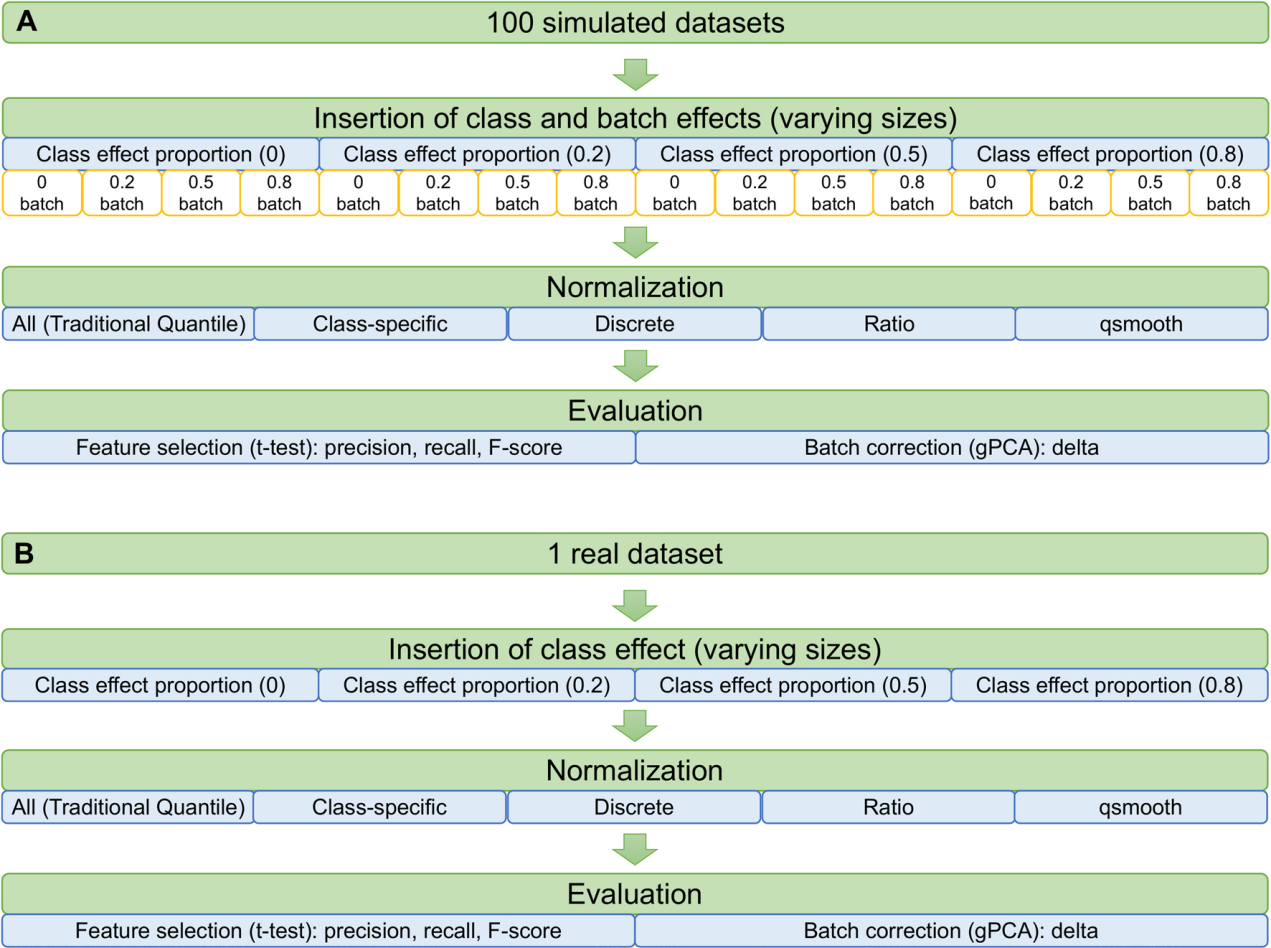
Simulation strategies for data with simulated class and batch effects (**A**) and data with real batch effects, but simulated class effects (**B**).

To simulate class-effect proportion (CEP), class effects are applied onto 0, 0.2, 0.5 and 0.8 of measured proteins (Fig. 2). The magnitude of applied effect sizes is randomly selected from 0.2, 0.5, 0.8, 1 and 2. The class effect is applied in one class, but not the other, and is a proportionate increment. For example, a 0.2 class-effect level means a 20% increment from the original value. When CEP is high, it leads to sample classes whose basal expression states are drastically different.

Batch effects are simulated similarly, except the batch effects are inserted according to batch factors (the categorization of technical batches). In this simplistic scenario, we simply assign half of the samples of each class, to each batch.

Since the set of differential variables are known a priori, normalization performance across the five strategies may be evaluated by statistical feature selection (based on the two-sample *t* test; α = 0.05 significance level) and overall batch-effect correction based on the gPCA delta^21^ (see below).

For statistical feature selection, the precision, recall and their harmonic mean (the F-score) are used. These are expressed as:

$$\begin{aligned} & {\text{Precision}} = { }\frac{TP}{{TP + FP}} \\ & {\text{Recall}} = { }\frac{TP}{{TP + FN}} \\ & {\text{F-score}} = 2 \times \frac{Precision \times Recall}{{Precision + Recall}} \\ \end{aligned}$$

where TP, FP and FN refer to true positives, false positives and false negatives, respectively. The efficacy of batch correction is evaluated using gPCA^21^. The gPCA delta measures the proportion of variance due to batch effects in test data, and is bound between 0 and 1. Ideally, we want this to be as low as possible following normalization.

### Evaluation using data with simulated class and batch effects

The D2.2 dataset is a one-class proteomics dataset (n = 8) derived from shotgun proteomics in a study of arctic squirrels with no technical replicates (batches)^22^. This dataset is largely free of any batch or class effects. As we have used it for a large variety of benchmarking experiments in the past, we would also be able to determine if the simulation outcomes from the various QN-strategies are compatible/unexpected with reference to previous studies. Batch and technical effects are simulated as described above and shown in Fig. 2A.

### Evaluation using data with real batch effects

The Renal Cancer Control dataset (designated RCC for ease of reference) is a one-class proteomics dataset (n = 4) with three technical replicates each (batches)^23^, making a total of 12 samples. This is a rare dataset because it is designed essentially for benchmarking. As with D2.2, we have also used it for a variety of benchmarking experiments in the past, and therefore we would also be able to determine if the simulation outcomes from across the various QN-strategies are consistent with respect to previous studies. Moreover, these samples all originate from normal kidney tissue and have already been well-characterized and described prior^24^.

To generate batch effects in RCC, two different batches are simply combined into one dataset (Fig. 2B). Since there is only one class in RCC, there are no real class effects. We therefore simulate class-effect proportions from 0 (negative control) to 0.8.

### Cross-analysis of multiple datasets normalized independently in the “combination” scenario

Many similar but small datasets are available online. To boost power, it is sometimes desirable to combine these datasets together as a form of “big-data” analysis (we term this the “combination” scenario). However, as constituent datasets are normalized independently; merely combining these can lead to the generation of batch effects, and reduce detectability of true biological signals.

**Figure 3:**
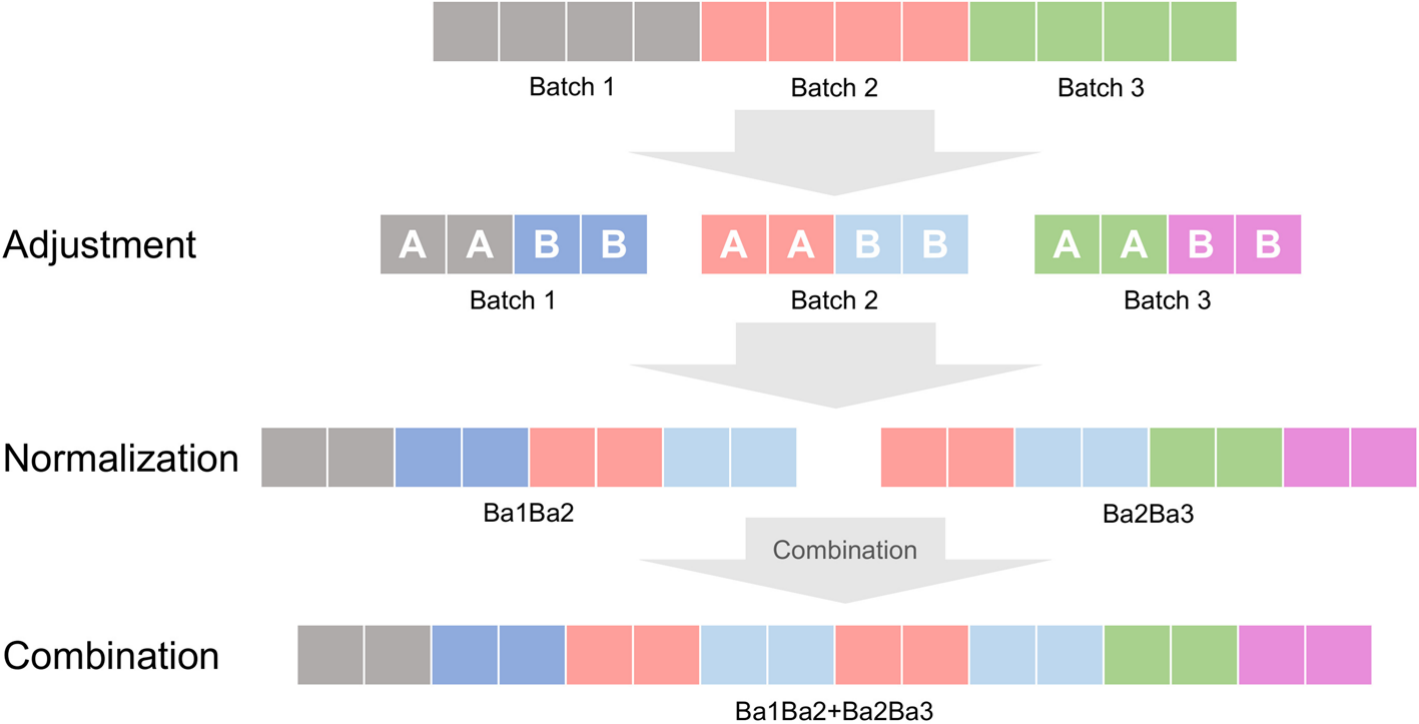
Cross analysis approach for analyzing discretely normalized datasets.

It is possible that amongst the various QN-strategies, some may have value in facilitating this “combination” scenario. RCC’s three natural technical replicates (1, 2 and 3) are used to identify the appropriate QN-strategy (Fig. 3).

We developed a 3-step procedure involving adjustment, normalization and combination (Fig. 3) to simulate the “combination” scenario. In adjustment, samples in each batch are assigned an arbitrary class label (A or B) and differential proteins assigned. In normalization, samples from 2 batches are assigned together such that we get two possible combinations, Batch1/Batch2 (Ba1Ba2) and Batch2/Batch3 (Ba2Ba3). The five QN-strategies are then deployed on these two combinations separately. In combination, the resulting Batch1/Batch2 and Batch2/Batch3 (Ba1Ba2 + Ba2Ba3) are combined, on which we then evaluate based on statistical feature selection and batch-effect measurements based on the gPCA delta^21^.

## Results

### The “All” quantile normalization strategy falls short

**Figure 4:**
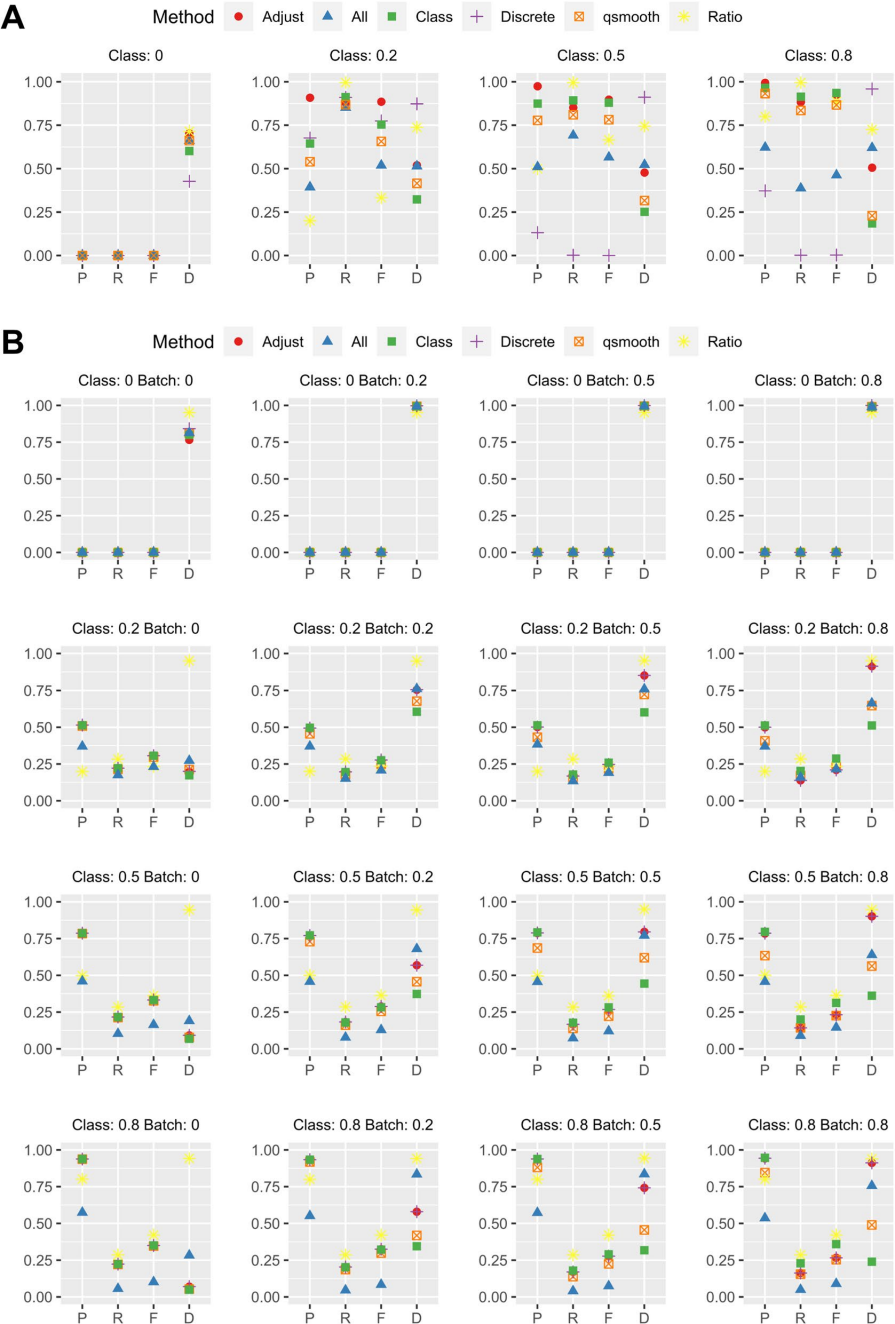
Performance of statistical feature selection (Precision: P, Recall: R and F-score: F) and batch effect correction (gPCA Delta: D) (**A**) statistical feature selection across the various quantile normalization strategies given increasing class effects (from 0 to 0.8). Data points shown here are the respective means across 100 simulations based on the RCC dataset. (**B**) Statistical feature selection across the various quantile normalization strategies given increasing class effects (from 0 to 0.8) and increasing batch effects (from 0 to 0.8). Data points shown here are the respective means across 100 simulations based on the D2.2 dataset. The “Adjust” scenario is not a quantile normalization strategy, it is the data after inserting class and/or batch effects, but no normalization.

We evaluate five QN (sub)strategies on two datasets, the RCC and D2.2. Since both datasets have effectively one class, we may randomly assign samples into arbitrary classes and simulate class effects by specifying which proteins should have a significant differential expression (see “Materials and methods” section). The proportion of proteins in the dataset which is designated differential is termed class-effect proportion (CEP). For reference, we also introduce the pre-adjustment scenario (“Adjust”), which is the form of data after insertion of class and batch-effects, but before normalization (Fig. 4).

Both RCC (natural batch effects, simulated class effects) and D2.2 (simulated batch effects, simulated class effects) demonstrate that the commonly used “All” quantile approach is generally poorer at signal recovery (Fig. 4).

In RCC, where a natural batch effect is created by combining technical replicates, the “All” quantile strategy performs generally the worst (in terms of F-scores) in statistical feature selection. Averaging across all CEPs evaluated, “All” is the second worst with an F-score of 0.52; “Class-specific” is the best at 0.86 (1.7-fold difference). Where CEP is low (0.20) and in what may be considered a typical scenario where “All” is expected to work well if not abysmally, “Discrete” is the best performing with an F-score of 0.77, and “All” second last at 0.52 (1.5-fold difference). When CEP is high (0.8) and what we consider to be a non-optimal scenario for “All”, “Class-specific” tops the list with an F-score of 0.94 while “All” performs very poorly with an F-score of 0.46 (2.0-fold difference). Expectedly, the greatest performance differential is in the scenario where CEP is high. But what is surprising is that “All” does not work well even in the low CEP scenario as well.

In the RCC evaluation, “All” is not always the worst. Other poorly performing strategies include the “Ratio” strategy, which is the worst in the small CEP scenario with an F-score of 0.33 (Fig. 4A; c.f. Table 1). Furthermore, we observe that “All” is moderate in its ability to downplay batch effects. Averaging across all CEPs evaluated, “All” ranks fourth with a gPCA Delta of 0.55, and the “Class-specific” approach ranks first with a Delta of 0.25 (2.0-fold difference). The performance ranking for batch-effect resistance is relatively stable for each CEPs examined.

**Table 1:** Rank distributions denoting performance based on statistical feature selection and batch effect correction using RCC data (c.f. Fig. 4A).

CEP	F-score				Delta			
	0	0.2	0.5	0.8	0	0.2	0.5	0.8
Adjust	0	**1**	**1**	2	5	3.5	3	3
Class-specific	0	3	2	**1**	2	**1**	**1**	**1**
qsmooth	0	4	3	3.5	3	2	2	2
All	0	5	5	5	4	3.5	4	4
Discrete	0	2	6	6	**1**	6	6	6
Ratio	0	6	4	3.5	6	5	5	5

The top ranked method is displayed in bold.

In RCC, the best approach for signal recovery, when CEP is small, is not to do anything (the “Adjust” scenario: pre-normalized data) where the F-score for “Adjust” is 0.89, compared to 0.77 for “Discrete”. However, “Class-specific” and “qsmooth” strategies catch up quickly as CEP increases (At a CEP of 0.8, the F-scores for “Class-specific” and “qsmooth” moves up to 0.94 and 0.87 respectively, where the F-score for “Adjust” is 0.93). This supports the notion that such strategies become increasingly suitable, when inter-class differences (higher CEP) become more pronounced.

D2.2 shows that the gPCA Delta is not always an objective measure of batch effects. It is unexpectedly high in cases where no batch effects are present with a mean value of 0.83 for all scenarios examined (Fig. 4B; Top left panel). Although once class effects are introduced, gPCA Deltas dropped to more congruent levels (between 0 and 0.25). Interestingly, the “Ratio” strategy seems to inflate batch effects such that gPCA Deltas are always high (no matter what the simulated CEP or batch level is). To demonstrate the correctness of the simulations, actual distribution values for D2.2 are shown in Supplementary Fig. S1.

Where statistical feature selection is concerned, D2.2 suggests that without batch effects (Fig. 4B; Leftmost column), as CEP increase from 0.2 to 0.8, the performance of the “All” strategy becomes progressively worse relative to other approaches: “All” has the lowest F-scores at 0.23 at a CEP of 0.2 and 0.10 at a CEP of 0.8, and also the second highest gPCA Delta with an average of 0.25 across all CEPs (after the “Ratio” strategy whose respective average Delta is 0.95). As CEP increases (from 0.2 onwards), all other strategies, including “Ratio”, outperform “All”.

Surprisingly, the “Ratio” strategy works very well for statistical feature selection although it also seems to preserve batch effects very strongly (Table 2). The higher statistical power is likely due to inflation of class effects as they are uniformly added to only samples from one class, such that a consistently high ratio would be obtained. Moreover, from a statistical point-of-view, it is actually inappropriate to produce arbitrary ratios for non-pairable data. In D2.2, the “Class-specific” approach has the highest precision at 0.75 (“All” is the worst at 0.46); second highest F-score at 0.30 (Ratio is the best at “0.33” and the worst is “All” at 0.14), but the lowest gPCA deltas with an average of 0.42 (“Ratio” is the worst at 0.95).

**Table 2:** Rank distributions denoting performance based on statistical feature selection and batch effect correction using D2.2 data (c.f. Fig. 4B).

Batch	F-score				Delta			
	0	0.2	0.5	0.8	0	0.2	0.5	0.8
**CEP: 0**								
Adjust	0	0	0	0	**1**	4	4	4
Class-specific	0	0	0	0	2	4	4	4
qsmooth	0	0	0	0	4	4	4	4
All	0	0	0	0	4	4	4	4
Discrete	0	0	0	0	4	4	4	4
Ratio	0	0	0	0	6	**1**	**1**	**1**
**CEP: 0.2**								
Adjust	2.5	2.5	2	4	2.5	4	4.5	4.5
Class-specific	2.5	2.5	2	**1**	**1**	**1**	**1**	**1**
qsmooth	2.5	2.5	5	4	4	2	2	2
All	5.5	6	5	4	5	4	3	3
Discrete	2.5	2.5	2	4	2.5	4	4.5	4.5
Ratio	5.5	5	5	4	6	6	6	6
**CEP: 0.5**								
Adjust	3	3	3	4	2.5	3.5	4.5	4.5
Class-specific	**3**	3	3	2	2.5	**1**	**1**	**1**
qsmooth	3	5	5	4	2.5	2	2	2
All	6	6	6	6	5	5	3	3
Discrete	3	3	3	4	2.5	3.5	4.5	4.5
Ratio	3	**1**	**1**	**1**	6	6	6	6
**CEP: 0.8**								
Adjust	3.5	4	4	3	2.5	3.5	3.5	4.5
Class-specific	3.5	4	2	3	2.5	**1**	**1**	**1**
qsmooth	3.5	4	4	5	2.5	2	2	2
All	6	6	6	6	5	5	5	3
Discrete	3.5	2	4	3	2.5	3.5	3.5	4.5
Ratio	**1**	**1**	**1**	**1**	6	6	6	6

The top ranked method is displayed in bold.

### Class-specific approaches are useful for combined analysis of independently normalized data

Due to limited sample availability and high running costs, many biological datasets are small and generally lack statistical power. Over time, many related biological datasets using similar high-throughput platforms have been published (albeit already processed and normalized before being shared online). A desirable analytical procedure is to take these datasets and combine them for the purpose of boosting power. This procedure is known as mega-analysis.

Since each dataset comes from a different source, combining these inevitably generates batch effects, which in turn, has adverse effects on statistical feature selection. Since RCC has three technical batches, we may use these in a combinatorial manner to demonstrate if any of the 5 QN-strategies has any value in preserving signal, while also being robust against any generated batch effects.

Using RCC, we first simulated class-effect proportion (CEP) from 0 to 0.8. Each technical batch in RCC with only class-effects simulated is termed as “Adjustment” phase data. We then simulated batch effects by combining the 3 technical batches derived from the “Adjustment” phase data using two scenarios, Ba1Ba2 and Ba2Ba3, which refer to Batches 1 and 2, and Batches 2 and 3 respectively. Ba1Ba2 and Ba2Ba3 are each normalized using the five QN-strategies. This post-normalized Ba1Ba2 and Ba2Ba3 are referred to as “Normalization” phase data. Finally, since we have two sets of normalized data due to Ba1Ba2 and Ba2Ba3, we combine these to create the “Combination” phase dataset.

**Figure 5:**
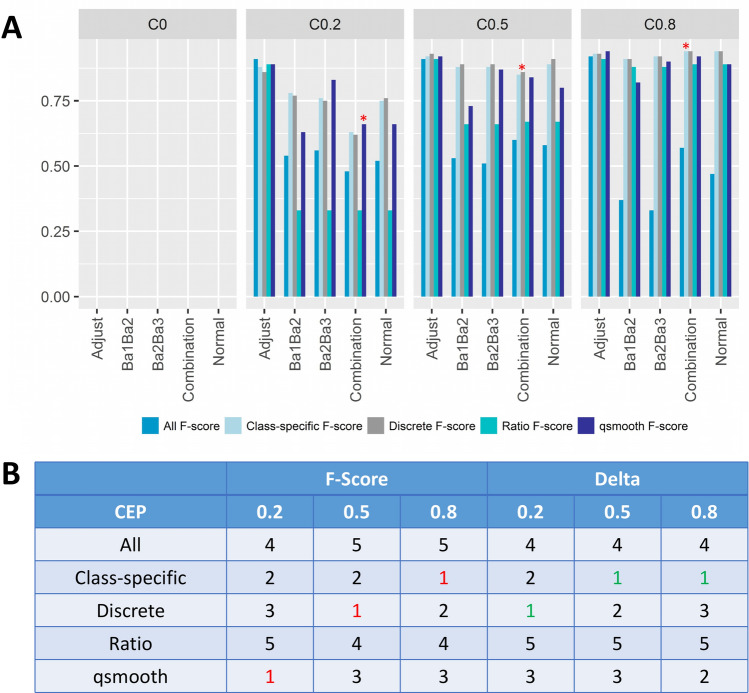
Feature-selection performance based on combining datasets that have been normalized separately given increasing class effect proportion (from 0 to 0.8) (**A**) distribution of F-scores Best performing method in the combination scenario is marked with a red *. (**B**) Feature-selection performance (F-Score) and Batch effect correction (Delta) based on the data combination scenario across 3 different class effect proportion (CEP) levels Performance values are summarized as ranks, where 1 is the best (highlighted in red or green), and 5 is the worst (c.f. Fig. 3).

For “Adjustment”, “Normalization” and “Combination” phase data, we perform statistical feature selection and summarize the findings as F-scores (Fig. 5A). We also evaluate changes in the measured batch effects based on the gPCA Delta (the actual values are detailed in Supplementary table S1). Furthermore, we summarize the findings as ranks in Fig. 5B for easy evaluation of performance for the “Combination” scenario.

When CEP is weak (0.2), there is overall degradation in statistical feature selection performance in the “Combination” phase. In the case of “All”, the F-scores for Ba1Ba2, Ba2Ba3 and “Combination” are 0.54, 0.56 and 0.48 respectively. This weaker performance in the “Combination” phase is also observed in the case of “Class-specific” for Ba1Ba2, Ba2Ba3 and “Combination” at 0.78, 0.76 and 0.63 respectively. Similar degradations are also observed for “Discrete” and “qsmooth”. Only “Ratio” remained invariant for Ba1Ba2, Ba2Ba3 and “Combination” phases, albeit with the same poor F-scores at 0.33.

These results may come across as unsurprising, since combining discretely normalized data (Ba1Ba2 + Ba2Ba3) will create additional batch effects that make it harder to select correct signal. Notably, the “Ratio” strategy performs the worst, followed by “All”. This is consistent with our earlier findings, since we already know that “Ratio” is the worst at dealing with batch effects (c.f. Fig. 4).

As CEP increases from 0.2 towards 0.8, all QN-strategies improve, including “Ratio”. For example, the F-scores for “Class-specific” and “Discrete” both increase to 0.94. However, the rate of improvement differs, with “All” moving slowly, and eventually languishing to become the worst-performing method with an F-score of 0.57 given a CEP of 0.8.

If we are working with multiple datasets with relatively low CEPs, the “qsmooth” approach is good. Here, the F-score for “qsmooth” is 0.66, with the next best performing method being “Class-specific” at 0.63. The worst is “Ratio” with an F-score of 0.33. However, if high CEPs are suspected to be present, then the “Class-specific” and “Discrete” strategies yield better results. Here, the F-score for “Class-specific” and “Discrete” is 0.94, with the next best performing method being “qsmooth” at 0.92.

When it comes to batch correction, the best strategies are “Discrete” and “Class-specific”, with “qsmooth” also being a good option (Fig. 5B). While “All” does not afford much adjustment for batch effects, the “Ratio” strategy is consistently the worst at batch-effect management (Fig. 5B; c.f. Fig. 4).

## Discussions

### Quantile normalization is affected by class-effect proportion and batch effects

Applying QN on a whole dataset assumes all samples in that dataset, irrespective of class, to have similar feature value (i.e. protein expression level) distributions. Given a dataset with two classes and a single batch. Suppose QN is applied blindly to the entire dataset, we will find that even when samples in the two classes have different feature value distributions, they are both normalized by QN to the same incorrect target feature value distribution (which is the average of the two true distributions weighted by the proportions of samples in the two classes). On the other hand, suppose QN is applied on the two classes of samples separately, the samples would be normalized to their respective target feature value distribution. Hence applying QN blindly to the entire dataset is less likely to produce good results than applying QN in a class-specific way (Supplementary Fig. S1).

Suppose further that there are two batches where QN is applied in a class- and batch-specific manner, four class-and batch-specific feature value distributions are produced for the four possible class-batch combinations. If the two batch-specific distributions for the same class are quite different, this makes it harder to correctly detect the actual differential features. On the other hand, suppose QN is applied in a class-specific way on the union of the two batches. Then the samples get normalized to the target feature value distribution of their respective class. This makes it easier to correctly detect the differential features than the class-and batch-specific application of QN.

However, there is an important caveat when QN is applied in a class-specific way to the union of batches: The feature value distribution of a class is the average of the feature value distribution of this class in the two batches weighted by the proportion of samples of this class in the two batches. This dependence on proportion of samples of the class in the two batches is likely to cause problems when QN is applied in this manner in multiple studies, and the normalized data from these studies are combined.

Despite its seemingly good properties, in our simulations for the traditional “All” QN-strategy, performing normalization on data where class-effect proportion (CEP) becomes progressively higher, generally leads to poorer identification of real signal (as evidenced by statistical feature selection evaluated on the F-score). This is further exacerbated by the presence of batch effects serving as an additional source of confounding. In contrast, for the other 4 QN strategies, as CEP increases, the F-score increases.

In practice, we are well aware that data normalization based on unreliable assumptions does more harm, by distorting the data, and creating invalid conclusions^16^. A very important erroneous assumption is that the different sample classes only involve few differential genes, and that in total, the overall distribution between samples should be similar, irrespective of class. These two assumptions are obviously invalid when comparing normal tissue against highly proliferative cancer tissue^17,18^.

Here, when CEP increases, applying quantile normalization on the whole dataset, is not effective, is error generating, and should be avoided.

### Use Class-specific quantile normalization when class-effect proportion is high and for “combination” scenarios

Amongst the five QN-strategies, across various class-effect proportion (CEP) and batch-effect levels, the “Class-specific” QN-strategy emerges as the victor. It is most effective when CEP is high. And so, based on both sets of simulations (D2.2 and RCC), we advocate the use of the “Class-specific” strategy, especially when class-effect proportion is high, and where there is also the presence of a strong batch effect (Fig. 4; Bottom right panel).

“Class-specific” QN also works well (relative to the other four strategies) as a normalization approach if the intended desire is to eventually combine these separate datasets in a bid for performing mega-analysis; that is, combining multiple separately-normalized datasets into one single whole for the purpose of enhancing power, signal recovery and functional analysis.

Although the focus here is methodological, there are many practical uses for “Class-specific” QN in the biological setting. As mentioned earlier, due to limited sample availability and high running costs, many biological datasets lack power. But combining these generates batch and/or other technical effects. If we want to more effectively leverage on biological data on which much resources (monetary, material and time) have been invested, doing normalization better can certainly help. In specific biomedical phenotypes, there are also diseases which clearly have high CEP. Cancer, being an obvious scenario suitable for “Class-specific” QN.

### Limitations of study

Although we evaluate five QN strategies, this study is essentially limited to proteomics data. Assuming platform-specific idiosyncratic bias is negligible, the outstanding issue of strong CEPs should manifest similarly in a non-platform specific manner. That is, we do not think the results would have differed greatly if we considered genomics or transcriptomics data.

However, it is true that different platforms have different traits (e.g. orders of magnitude amongst measured variables, the number of measurable variables, different technical effects), which may affect the relative performance of statistical feature selection or batch-effect measurement. A future direction of this study would be to investigate if there are discernible differences across platforms, and if our finding that the “Class-discrete” strategy is optimal when CEP is strong, still holds true.

Other limitations are simulation and measurement of batch effects. The approach for simulating batch effects here assumes uniformity, such that every protein carries some information regarding batch effects and is multiplicative. However, techniques for non-uniform or additive batch simulation also exist^19^ and could be considered as additional proof regarding the batch-effect robustness manifested by some of the strategies tested here. Finally, while very convenient, the gPCA Delta does not appear to be always stable or objective, and in the absence of class effects, may manifest as high (close to 1, indicating presence of batch effects), even when no true batch effects actually exist. However, this is a compromise, as a summary statistic measuring total batch effect is still more desirable than to check for batch effects manually, via the hundreds of scatterplots that may be generated per simulation.

Finally, this study is based essentially on simulations, with assumptions on how batch and class effects may manifest. While it is possible to deploy “Class-specific” QN on real data, it is much harder to prove that the predictions are necessarily correct. This would require experimental validation and mechanistic studies, which goes beyond the scope of this study.

## Conclusions

Quantile normalization is often used to normalize –omics datasets. If inter-class effect proportion and/or batch-effects are strong, then a careless but commonly used “blind” approach which applies quantile normalization on the entire dataset is wrong, and leads to poorer statistical feature selection while also not sufficiently addressing batch effects (if present). Fortunately, this is easily addressed by alternative strategies, namely, those that take into account class-proportion effects. Here, we demonstrate that such strategies, including the “Class-specific” and “qsmooth” approaches, readily outperform the blind approach to quantile normalization; and they are also robust, preserving useful signal even when considering multiple independently-normalized datasets.

## Supplementary information

Supplementary Information.

## Data Availability

All data analysed in this study are available in the GitHub repository (https://github.com/gohwils/NetProt/releases/tag/0.1/Data.zip).
