# Supplementary material for: How to do quantile normalization correctly for gene expression data analyses
Source: Sci Rep. 2020 Sep 23;10:15534. doi: 10.1038/s41598-020-72664-6 (PMC7511327; doi:10.1038/s41598-020-72664-6)
Supplement: Supplementary file 1 — Supplementary Information. [file 41598_2020_72664_MOESM1_ESM.docx]

**How to do quantile normalization correctly for gene expression data analyses**

Yaxing Zhao^1^, Limsoon Wong^2,3^, Wilson Wen Bin Goh^4*^

1. School of Pharmaceutical Science and Technology, Tianjin University, China
2. Department of Computer Science, National University of Singapore, Singapore
3. Department of Pathology, National University of Singapore, Singapore
4. School of Biological Sciences, Nanyang Technological University, Singapore

*Corresponding Author(s): Wilson Wen Bin Goh

School of Biological Sciences, Nanyang Technological University, 60 Nangyang Drive, Singapore 637551; Tel: +65 63162800, Fax: +65 6791 3856;

Email: wilsongoh@ntu.edu.sg, [goh.informatics@gmail.com](mailto:goh.informatics@gmail.com)

# Supplementary Table

Table S1 Statistical feature selection (F-score) and batch effect correction (Delta) performance

|  | All | | | | | Class-specific | | | | Discrete | | | | Ratio | | | | qsmooth | | | |
| --- | --- | --- | --- | --- | --- | --- | --- | --- | --- | --- | --- | --- | --- | --- | --- | --- | --- | --- | --- | --- | --- |
|  | Method | Precision | Recall | F-score | Delta | Precision | Recall | F-score | Delta | Precision | Recall | F-score | Delta | Precision | Recall | F-score | Delta | Precision | Recall | F-score | Delta |
| C0 | Adjust | 0.00 | 0.00 | 0.00 | 0.70 | 0.00 | 0.00 | 0.00 | 0.70 | 0.00 | 0.00 | 0.00 | 0.70 | 0.00 | 0.00 | 0.00 | 0.70 | 0.00 | 0.00 | 0.00 | 0.70 |
|  | Ba1Ba2 | 0.00 | 0.00 | 0.00 | 0.52 | 0.00 | 0.00 | 0.00 | 0.50 | 0.00 | 0.00 | 0.00 | 0.52 | 0.00 | 0.00 | 0.00 | 0.66 | 0.00 | 0.00 | 0.00 | 0.52 |
|  | Ba2Ba3 | 0.00 | 0.00 | 0.00 | 0.21 | 0.00 | 0.00 | 0.00 | 0.19 | 0.00 | 0.00 | 0.00 | 0.36 | 0.00 | 0.00 | 0.00 | 0.56 | 0.00 | 0.00 | 0.00 | 0.20 |
|  | Combination | 0.00 | 0.00 | 0.00 | 0.64 | 0.00 | 0.00 | 0.00 | 0.65 | 0.00 | 0.00 | 0.00 | 0.35 | 0.00 | 0.00 | 0.00 | 0.76 | 0.00 | 0.00 | 0.00 | 0.64 |
|  | Normal | 0.00 | 0.00 | 0.00 | 0.66 | 0.00 | 0.00 | 0.00 | 0.60 | 0.00 | 0.00 | 0.00 | 0.42 | 0.00 | 0.00 | 0.00 | 0.71 | 0.00 | 0.00 | 0.00 | 0.66 |
| C0.2 | Adjust | 0.91 | 0.92 | 0.91 | 0.52 | 0.91 | 0.88 | 0.88 | 0.54 | 0.90 | 0.83 | 0.86 | 0.54 | 0.91 | 0.89 | 0.89 | 0.48 | 0.91 | 0.88 | 0.89 | 0.53 |
|  | Ba1Ba2 | 0.42 | 0.84 | 0.54 | 0.44 | 0.73 | 0.86 | 0.78 | 0.29 | 0.75 | 0.81 | 0.77 | 0.32 | 0.20 | 0.99 | 0.33 | 0.66 | 0.55 | 0.80 | 0.63 | 0.35 |
|  | Ba2Ba3 | 0.46 | 0.82 | 0.56 | 0.23 | 0.67 | 0.86 | 0.76 | 0.16 | 0.69 | 0.83 | 0.75 | 0.33 | 0.20 | 0.99 | 0.33 | 0.60 | 0.87 | 0.82 | 0.83 | 0.14 |
|  | Combination | 0.33 | 0.92 | 0.48 | 0.52 | 0.47 | 0.94 | 0.63 | 0.40 | 0.47 | 0.92 | 0.62 | 0.32 | 0.20 | 1.00 | 0.33 | 0.78 | 0.52 | 0.91 | 0.66 | 0.42 |
|  | Normal | 0.38 | 0.89 | 0.52 | 0.52 | 0.64 | 0.91 | 0.75 | 0.34 | 0.67 | 0.88 | 0.76 | 0.40 | 0.20 | 0.99 | 0.33 | 0.75 | 0.54 | 0.87 | 0.66 | 0.41 |
| C0.5 | Adjust | 0.97 | 0.86 | 0.91 | 0.46 | 0.98 | 0.88 | 0.92 | 0.48 | 0.98 | 0.90 | 0.93 | 0.44 | 0.98 | 0.88 | 0.91 | 0.47 | 0.98 | 0.88 | 0.92 | 0.46 |
|  | Ba1Ba2 | 0.50 | 0.63 | 0.53 | 0.48 | 0.91 | 0.86 | 0.88 | 0.18 | 0.93 | 0.88 | 0.89 | 0.17 | 0.50 | 0.99 | 0.66 | 0.65 | 0.74 | 0.76 | 0.73 | 0.24 |
|  | Ba2Ba3 | 0.50 | 0.60 | 0.51 | 0.29 | 0.89 | 0.87 | 0.88 | 0.12 | 0.90 | 0.89 | 0.89 | 0.40 | 0.50 | 0.99 | 0.66 | 0.60 | 0.96 | 0.82 | 0.87 | 0.13 |
|  | Combination | 0.51 | 0.78 | 0.60 | 0.55 | 0.78 | 0.94 | 0.85 | 0.30 | 0.79 | 0.95 | 0.86 | 0.35 | 0.50 | 1.00 | 0.67 | 0.78 | 0.80 | 0.89 | 0.84 | 0.32 |
|  | Normal | 0.51 | 0.72 | 0.58 | 0.53 | 0.88 | 0.91 | 0.89 | 0.23 | 0.89 | 0.93 | 0.91 | 0.39 | 0.50 | 1.00 | 0.67 | 0.75 | 0.78 | 0.84 | 0.80 | 0.29 |
| C0.8 | Adjust | 0.99 | 0.88 | 0.92 | 0.47 | 0.99 | 0.89 | 0.93 | 0.49 | 0.99 | 0.89 | 0.93 | 0.47 | 0.99 | 0.87 | 0.91 | 0.51 | 0.99 | 0.90 | 0.94 | 0.48 |
|  | Ba1Ba2 | 0.57 | 0.29 | 0.37 | 0.52 | 0.98 | 0.87 | 0.91 | 0.10 | 0.98 | 0.87 | 0.91 | 0.13 | 0.80 | 0.99 | 0.88 | 0.66 | 0.91 | 0.78 | 0.82 | 0.14 |
|  | Ba2Ba3 | 0.52 | 0.25 | 0.33 | 0.28 | 0.97 | 0.88 | 0.92 | 0.11 | 0.97 | 0.88 | 0.92 | 0.46 | 0.80 | 0.99 | 0.88 | 0.58 | 0.99 | 0.85 | 0.90 | 0.10 |
|  | Combination | 0.69 | 0.51 | 0.57 | 0.62 | 0.93 | 0.95 | 0.94 | 0.23 | 0.94 | 0.94 | 0.94 | 0.39 | 0.80 | 1.00 | 0.89 | 0.77 | 0.94 | 0.91 | 0.92 | 0.25 |
|  | Normal | 0.63 | 0.40 | 0.47 | 0.61 | 0.97 | 0.92 | 0.94 | 0.17 | 0.97 | 0.92 | 0.94 | 0.43 | 0.80 | 1.00 | 0.89 | 0.73 | 0.94 | 0.86 | 0.89 | 0.21 |

# Supplementary Figure


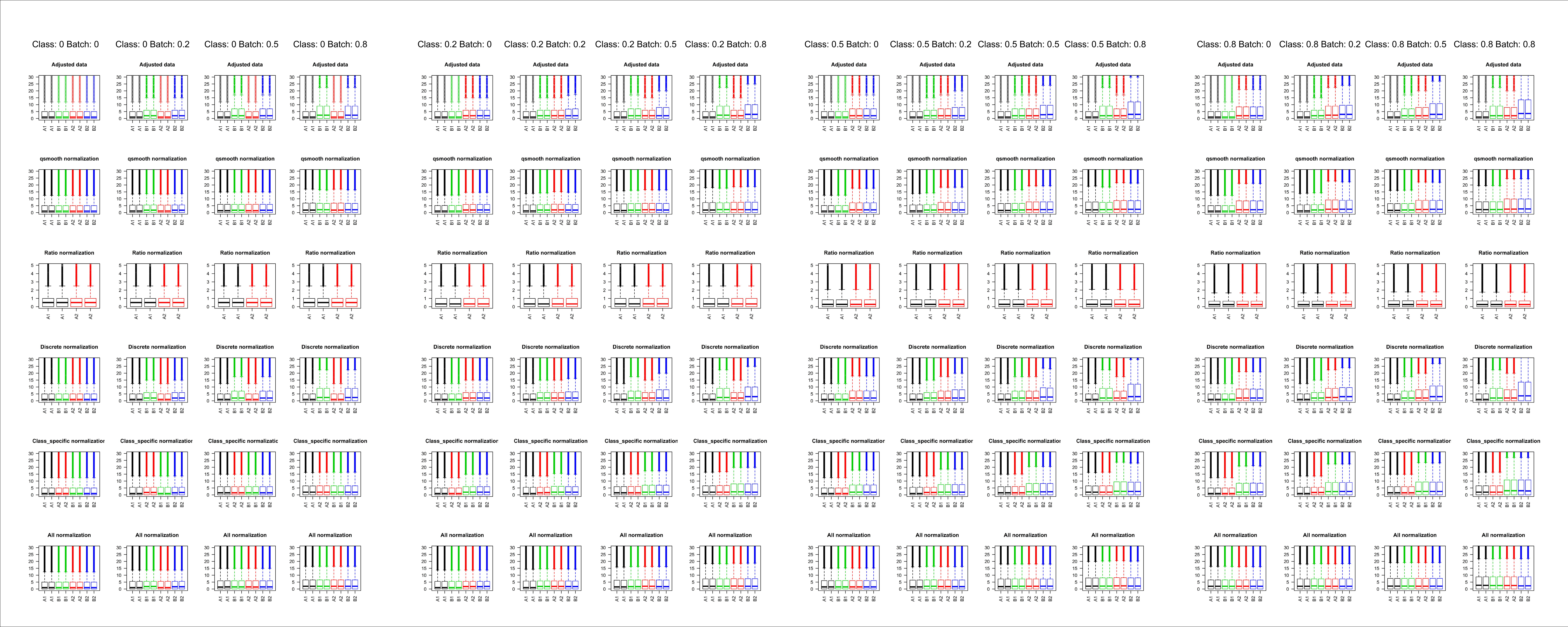


Figure S1 Boxplots showing distributions of data in D2.2 for various batch effect and class effect proportion simulations
